# Facile Regulation of Shell Thickness of the Au@MOF Core-Shell Composites for Highly Sensitive Surface-Enhanced Raman Scattering Sensing

**DOI:** 10.3390/s22187039

**Published:** 2022-09-17

**Authors:** Boen Li, Yaling Liu, Jie Cheng

**Affiliations:** Institute of Quality Standards and Testing Technologies for Agro-Products, Chinese Academy of Agricultural Sciences, Beijing 100081, China

**Keywords:** core-shell composites, MOF, SERS, sensing, structural regulation

## Abstract

Metal-organic frameworks (MOFs)-based core-shell composites have advanced the development of surface-enhanced Raman scattering (SERS) analysis, which originates from the promising structural characteristics of the outer framework material as well as the inherent plasmonic properties of the novel metal structure core (for example, nanoparticle, MNP). However, the SERS effect only exists directly in the surface of MNP or restricted around the plasmonic MNP surface. Consequently, the nanoscale control of the thickness of MOF shell in hybrid core-shell substrates is highly desirable. Despite the great effects which have been made to integrate various MOF matrices with MNP for the purpose of improving the SERS activity, the nanoscale thickness control of MOF shell remains a significant challenge. Here, we report a facile regulation method that enables the Au NP to be encapsulated by a zirconium-based MOF (BUT-17) with different thickness through the controlling of synthesis parameters. This method provides a promising strategy for optimizing the activity of core-shell SERS substrates for potential trace detection.

## 1. Introduction

In advancing the development of surface-enhanced Raman scattering (SERS) analysis technology, multi-component nanostructures such as core-shell nanoparticles (NPs) have made great progress and attracted considerable attention. Integrating the stabilizing matrix of polymers, mesoporous silica, and transition-metal materials with plasmonic noble metal NP (NMNP) is a common strategy to promote SERS performance [1,2,3], which is derived from the inherent plasmonic properties of NMNP and is motivated by the targeted chemical capturing of analytes through surface-grafted external materials to localize them on plasmonic surfaces [4]. In particular, metal-organic frameworks (MOFs) are the most reported encapsulation materials. Based on their preeminent structural characteristics, such as having a highly porous crystalline structure, extremely high surface area, and tunable pore size, MOFs serves as the protective shell to prevent the aggregation of MNPs while selectively preconcentrating the target compound in close proximity to the surface of the NP core, featuring a core-shell SERS platform with unprecedented performance of highly sensitive and selective SERS sensing [5,6]. Although the Raman signal of the target compound can be enhanced by many orders of magnitude, the SERS effect only exists directly in the surface of NMNP (described as contact-mode SERS) or restricted around the plasmonic NMNP surface (normally less than 5 nm) based on the long-range electromagnetic (EM) enhancement effect, which is mainly because of the exponential decay of the electric field with the nanoscale distance between the target molecule and NMNP surface [7]. Consequently, to achieve high SERS activities, nanoscale control of the thickness of MOF shell in hybrid core-shell substrates is highly desirable.

Herein, we report a regulation route that allows the thickness of MOF shell to be controlled effectively during one-pot synthesis of core-shell substrates. A new type of Au @ Zr-based MOF (namely BUT-17) composite was prepared as the basic core-shell structure for investigation. The regulation strategy mainly involves the control of key reaction conditions during the synthesis process, such as the reaction temperature, the added amount of the Au cation (HAuCl_4_), the used of metal salt center (ZrCl_4_) and organic linker (tricarboxylic acid-based ligand (5′(4-carboxyphenyl)-[1,1′:3′,1″-terphenyl]-3,4″,5-tricarboxylic acid, H_4_CPTTA)). Subsequently, tetramethyl-thiuram disulfide (thiram), a familiar pesticide compound, was chosen as the Raman probe for the investigation of shell thickness-dependent SERS activity. Furthermore, with the help of developed regulation strategy, the core-shell Au@BUT-17 substrates were optimized to a suitable shell thickness for sensitive SERS recognition of ten different target analytes, including the representative dioxin compounds (2,3,7,8-tetrachlorodibenzo-p-dioxin, TCDD and 3,3′,4,4′-Tetrachlorobiphenyl, PCB-77), an emerging bisphenol molecule (1,1-bis(4-hydroxyphenyl)ethane, BPE), and the familiar antibiotic residue (tetracy-cline, doxycycline, chlortetracycline, ciprofloxacin, enrofloxacin, sulfamethazine, and sulfacetamide). The work provided a rational route and alternative scientific tool towards the optimizing of core-shell hybrid substrates for highly sensitive SERS detection.

## 2. Materials and Methods

### 2.1. Reagents and Materials

Chloroauric acid tetrahydrate (HAuCl_4_·4H_2_O, >47.8%), formic acid, methanol, and ethanol with the analytical grade were purchased from the Sinopharm Chemical Reagent Co., Ltd., Shanghai, China, N,N-dimethylformamide (DMF) was obtained from ANPEL Laboratory Technologies (Shanghai) Inc., Shanghai, China, Polyvinylpyrrolidone (PVP, Mw 40,000) were purchased from Solarbo Life Sciences Inc. (Beijing, China). Tetramethylthiuram disulfide (thiram) (100 mg, Dr. Ehrenstorfer) was selected as model probed molecule, which was purchased from J&K scientific Ltd. (Beijing, China) and the corresponding standard solutions (*C* = 100 ng·mL^−1^) were diluted with methanol. Two representative dioxin compounds (2,3,7,8-tetrachlorodibenzo-p-dioxin, TCDD and 3,3′,4,4′-Tetrachlorobiphenyl, PCB-77) were prepared with methanol (*C* = 10 ng·mL^−1^). The analysis solution of 1,1-bis (4-hydroxyphenyl) ethane (BPE, *C* = 100 μg·mL^−1^, methanol) was purchased from J&K scientific Ltd. (Beijing, China) and diluted to the certain concentration (*C* = 1 μg·mL^−1^) with methanol solution. Seven kinds of antibiotic residues (tetracycline, doxycycline, chlortetracycline, ciprofloxacin, enrofloxacin, sulfamethazine, and sulfacetamide) were prepared with methanol solution (*C* = 1 μg·mL^−1^).

### 2.2. One-Pot Synthesis of Core-Shell Au@BUT-17 NPs

The organic ligands of BUT-17 (5′-(4-carboxyphenyl)-[1,1′:3′,1″-terphenyl]-3,4″,5-tricarboxylic acid, H_4_CPTTA) were synthesized as reported previously [8]. Then, 8 mL of DMF-Ethanol (*v*:*v* = 5:3) mixed solution containing 6 mg of ZrCl_4_, 5 mg of H_4_CPTTA, and 0.8 g of PVP were thoroughly mixed in a 20 mL glass vial by vortexing for 1 min. After mixing, 300 μL of formic acid was injected into the mixture followed by the addition of 400 μL of 1 wt % HauCl_4_ solution. The reaction mixture was left undisturbed at 120 °C for 3 h. The products were collected by centrifugation at 4000 rpm for 4 min, washed three times each with water and methanol. Finally, the obtained Au@BUT-17 NPs was resolved in 4 mL of methanol for further characterization and SERS detection.

### 2.3. Regulation of the Thickness of MOF Shell in Au@BUT-17 NPs

The key reaction conditions, such as the reaction temperature, the added amount of HauCl_4_, the used of ZrCl_4_ and H_4_CPTTA were regulated during the synthesis process. The details were provided in Supplementary Materials (Tables S1–S5).

### 2.4. Characterization

Transmission electron microscopy (TEM) images were obtained on a JEM-2100F (JEOL) instrument with annular dark field (ADF) detectors, operating at 200 kV, with the samples deposited on carbon-coated copper grids. Energy dispersive X-ray (EDX) elemental mapping was characterized by energy-dispersive spectroscopy system (X-MaxN 80T, Oxford Instruments, Abingdon, UK). X-ray photoelectron spectroscopy (XPS) investigation was recorded on the AXIS Supra spectrometer (SHIMADZU, Kyoto, Japan) using a monochromatic Al Kα X-ray source. Nitrogen sorption isotherms were measured at 77 K with gas sorption analyzer ASAP-2460 (Micromeritics, Norcross, GA, USA).

### 2.5. SERS Measurement and Data Analysis

Next, 500 μL of the sample were mixed with 500 µL Au@HOF-20 NPs thoroughly in sample vials (12 × 32 mm, Thermo scientific Co., Ltd., Shanghai, China) for 15 min. SERS measurements were collected at a portable Raman spectrophotometer (Raman Tracer-200-HS, OptoTrace Technologies, Inc., Sunnyvale, CA, USA) at 200 mW laser power and 10 s exposure time with an incident laser wavelength of 785 nm. Each spectrum was an average of two scans in the spectral range of 250 cm^−1^ to 2250 cm^−1^. Gaussian smoothing was performed in the raw Raman spectra to reduce the spectral noise. Second derivative transformation and polynomial substrate were also used. The SERS spectra were plotted with Origin Pro 9.1 software (Origin Lab, Northampton, MA, USA).

## 3. Results and Discussion

### 3.1. Synthesis and Characterizations

**Figure 1:**
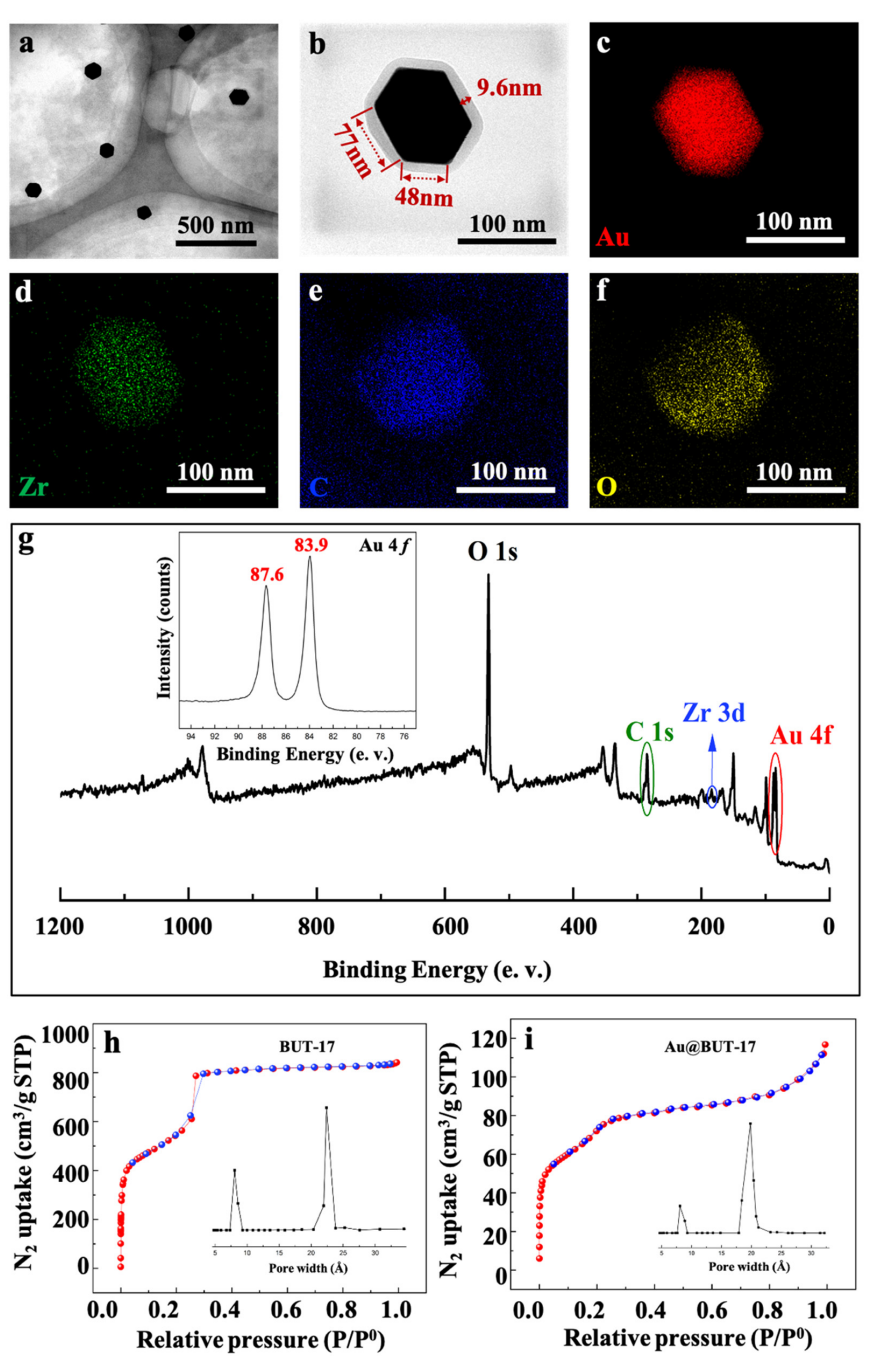
(**a**) TEM image of Au@BUT-17 NPs synthesized; (**b**) representative TEM image of single Au NP with diameter 48 nm × 77 nm encapsulated in BUT-17 shell with the thickness of 9.6 nm; (**c**–**f**) EDX elemental mapping of the element Au, Zr, C, and O shown in (**b**); (**g**) XPS spectrum of Au@BUT-17 NPs and the enlarged Au 4f region (insert); (**h**,**i**) N_2_ adsorption/desorption isotherms of BUT-17 and Au@BUT-17 at 77 K. The inset shows DFT pore size distribution evaluated by using the N_2_ adsorption data.

For the fabrication of core-shell Au@BUT-17 structure, the one-pot synthesis method was performed by mixing polyvinyl pyrrolidone (PVP) and the precursors of both AuNP and BUT-17 (HAuCl_4_, ZrCl_4_, and H_4_CPTTA) in reaction solution containing N,N-dimethylformamide (DMF) and ethanol. During the synthesis process DMF, ethanol, and PVP are found indispensable to the formation of core-shell structural unit. DMF is the reducing agent for the synthesis Au core [9], while ethanol boosts the MOF which preferentially grows onto the Au core, resulting in the formation of core-shell composites [10]. No core-shell NPs were obtained in absence of ethanol (Figure S1a). PVP is used for preventing AuNPs from aggregation. The aggregation between Au NPs preferentially takes place without the presence of PVP as soon as the formation of Au NPs (Figure S1b) [11]. The morphological characterization of the synthetic core-shell Au@BUT-17 NPs are presented in Figure 1a. The thickness of shell is (8.8 ± 1.3) nm, while the edge length of hexagonal-shaped Au core is (51.0 ± 6.8) nm × (79.0 ± 7.2) nm from the top view. The composition of the representative core-shell NP shown in Figure 1b is evidenced by high-angle annular dark field scanning transmission electron microscopy (HADDF-STEM). The energy-dispersive X-ray (EDX) elemental mapping clearly shows that the element Au is located in the center of core-shell NP, while the element Zr, C, and O of BUT-17 are homogenously distributed throughout the whole outer shell (Figure 1c–f). The X-ray photoelectron spectroscopy (XPS) characterization of Au@BUT-17 NPs also shows the presence of oxygen, carbon, Au, and Zr element (Figure 1g). The Au 4f peaks located at 83.9 eV (4f 7/2) and 87.6(4f 5/2) eV can be observed (Figure 1g, inset). As the MOF shell grows, the Brunauer-Emmett-Teller (BET) surface area of the Au@BUT-17 NPs decreases from 2221.87 m²·g^−1^ to 256.42 m²·g^−1^, when compared with the pristine BUT-17 material (Figure 1h,i). This is mainly due to the contribution of AuNP core to the mass of the core-shell NPs. The corresponding pore size of as-prepared core-shell NPs was calculated as two types of pores of 8.1 Å and 19.8 Å by DFT methods, being close to pure MOF materials (8.6 Å and 22.4 Å). The results showed that the encapsulation process does not destroy the original structure of outer BUT-17 shell.

### 3.2. Regulation of the Thickness of Shell in Core-Shell Composites

Not only by promoting the formation of core-shell NPs, the control of these synthesis parameters is found to be a feasible strategy to regulate the thickness of shell in core-shell composites. The effect of reaction parameters such as the reaction temperature, quantity of HAuCl_4_, and the amount of MOF precursors was investigated in detail.

**Figure 2:**
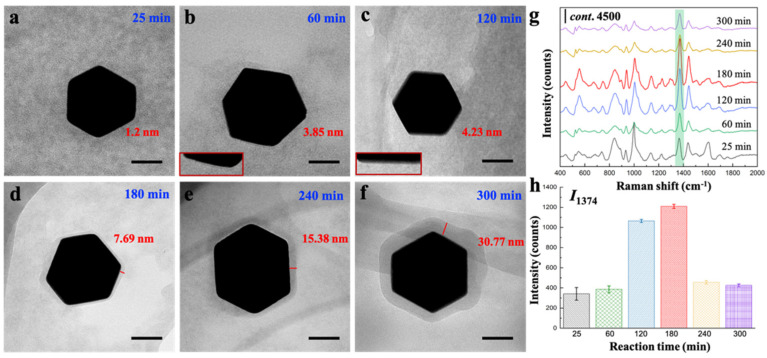
(**a**–**f**) Representative TEM images of core-shell Au@BUT-17 NP with increased shell thickness. Scale bar: 50 nm; (**g**) SERS spectra of thiram; and (**h**) the comparison of I_1374_ in the presence of Au@BUT-17 prepared in different reaction time ranged from 25 min to 300 min.

Firstly, we can adjust the reaction time (t) to control the growth of MOF shell. The t-dependent shell thickness experiments were performed by varying the reaction time while fixing other prepared conditions (Table S1). As-synthesized core-shell Au@BUT-17 NPs with the coating thickness of 1.2 nm, 3.85 nm, 4.23 nm, 7.69 nm, 15.38 nm, and 30.77 nm were obtained (Figure 2a–f; Figure S2) when t-values were set as 25, 60, 120, 180, 240, and 300 min, respectively. The results show that the shell thickness was significantly correlated with t. The crystallization of outer MOF fraction can be prompted by increasing the reaction time while the size of Au core remains similar. The thickness-dependent SERS performance was evaluated by comparing the SERS activity towards the probed thiram compound in the presence of Au@BUT-17 substrates with different shell thickness (Figure 2g). When the MOF thickness increased from 1.2 nm to 7.69 nm, the intensity of characteristic peak located at 1374 cm^−1^ (I_1374_) become stronger, demonstrating increased SERS activity until reaching to the peak value. One plausible explanation is that a shell too thin is subject to the insufficient capture for target compound, resulting in fewer molecules to be detected, which was consistent with the previous study [12]. However, the further increasement of thickness was not beneficial for improving the SERS activity. It was evidenced by the decreasing of I_1374_ with the thickness increased from 7.69 nm to 30.77 nm, corresponding with the prolonging of t from 180 min to 300 min. It is indicated that the decay of local field with the distance from the particle surface still exists, despite the high dielectric constant of MOF increases the penetration depth of electromagnetic field. Greater shell thickness possibly leads to the random location of thiram molecule in the area far away from the Au surface.

**Figure 3:**
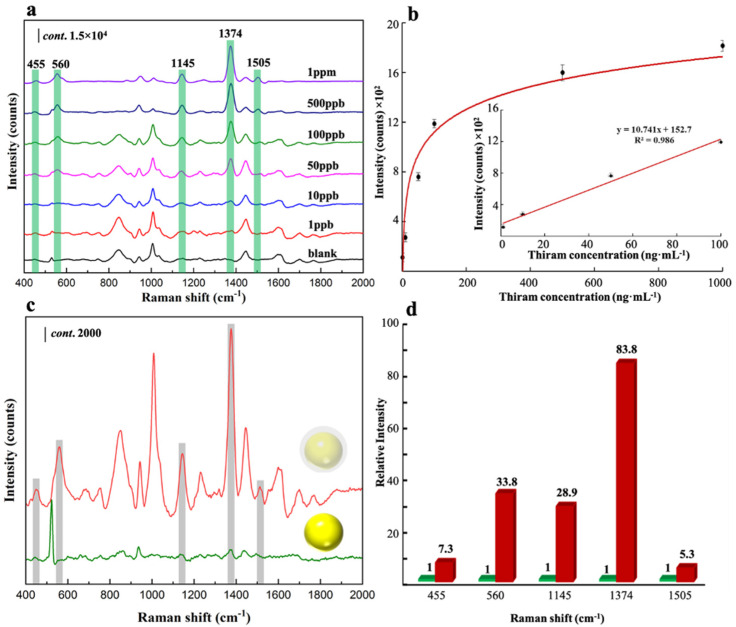
(**a**) Thiram concentration-dependent SERS spectra; (**b**) plot of Raman intensity (I_1374_) as a function of thiram concentration from 1 to 1000 ng·mL^−1^. The inset shows the linear response for thiram range of 1 to 100 ng·mL^−1^; (**c**) SERS spectra of thiram in the presence of Au NP and Au@BUT-17; (**d**) the relative Raman intensity of characteristic peaks located at 455 cm^−1^, 560 cm^−1^, 1145 cm^−1^, 1374 cm^−1^ and 1505 cm^−1^.

Au@BUT-17 NPs with the shell thickness of 7.69 nm, which demonstrated the highest SERS activity, was selected for the following investigation of SERS performance. The SERS spectra of thiram with concentrations ranged from 1 ng·mL^−1^ to 1000 ng·mL^−1^ were collected in Figure 3a. Five characteristic peaks of thiram were clearly identified at 455 cm^−1^, 560 cm^−1^, 1145 cm^−1^, 1374 cm^−1^ and 1505 cm^−1^, which were ascribed to δ(CSS) coupled with δ(CNC), υ(SS), ρ(CH_3_) coupled with υ(CN), δ(CH_3_), and υ(CN), respectively [13,14]. As the concentration of thiram increases, the multiple fingerprint peaks gradually increase. The peak intensity located at 1374 cm^−1^ (I_1374_) was selected for quantitative calculation. I_1374_ increased along with the increment of thiram concentrations from 1 ng·mL^−1^ to 1000 ng·mL^−1^ (Figure 3b). A good linear response was obtained in the concentration range of 1 ng·mL^−1^ to 100 ng·mL^−1^ (Figure 3b, inset), with the linear regression equation is y = 10.741x + 152.7, and the correlation coefficient (R^2^) of 0.986. The limit of detection (LOD) was estimated as low as 0.05 ng·mL^−1^ based on a signal-to-noise of 3 (S/N = 3). The highly sensitive and wide quantitation range demonstrate the excellent SERS performance, which feature the excellent structural property of highly ordered porous MOF shell. Compared with bare plasmonic Au NPs (Figure S3) the Au@BUT-17 hybrids demonstrate obviously better SERS activity towards the recognition of thiram (100 ng·mL^−1^) (Figure 3c,d). The surface area as high as 256.42 m²·g^−1^ (Figure 1i) provides readily accessible adsorption sites to capture thiram molecules in solution. The host-guest interaction between probed thiram and secondary active sites originate from nanochannels of MOF promote the analytical thiram access onto the plasmonic MNP surface. The near-field EM enhancement makes a significant contribution to the overall SERS mechanism [15].

**Figure 4:**
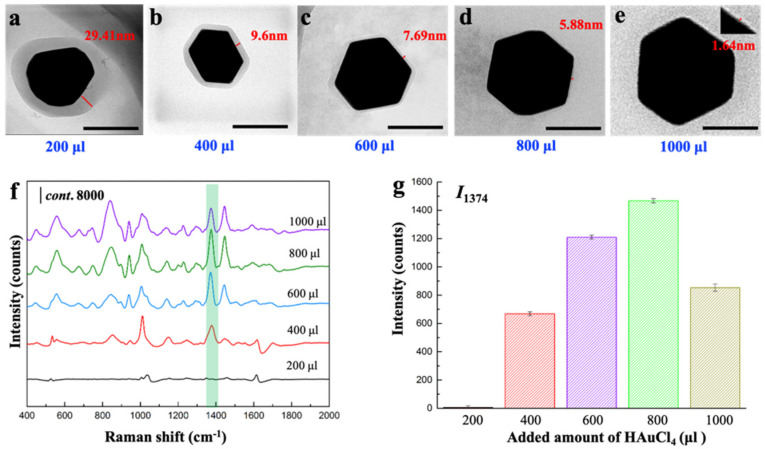
(**a**–**e**) Representative TEM images of core-shell Au@BUT-17 NP prepared with increased amount of HAuCl_4_ (μL). Scale bar: 100 nm; (**f**) SERS spectra of thiram; and (**g**) the comparison of I_1374_ in the presence of Au@BUT-17 prepared in different amounts of HAuCl_4_ ranged from 200 μL to 1000 μL.

Secondly, the added amount of Au precursors can indirectly change the shell thickness. During the synthesis, we varied the amounts of HAuCl_4_ (1%, *w*/*v*) as 200, 400, 600, 800 and 1000 μL while fixing the other conditions (Table S2). The obtained core-shell NPs demonstrated the decreased shell thicknesses as 29.41, 9.6, 7.69, 5.88 and 1.64 nm, respectively (Figure 4a–e, Figure S4). Thanks to the rapid formation of Au NP core compared with the growth of MOF shell, the more HAuCl_4_ were added, the larger Au NP cores were obtained. Correspondingly, the formation of MOF shells is thinner in the produced core-shell composites when fixing the usage of MOF precursors. According to the previous study [16], with the decreasing of shell thickness, the SPR peak of AuNPs red shifts while the SPR peak intensity increases. Thus, the SERS activity could be improved. This was consistent with our results. As shown in Figure 4f, SERS spectra of thiram clearly demonstrated the shell thickness-dependent sensitivity of SERS detection. As the amounts of HAuCl_4_ increased from 200 μL to 800 μL, SERS activity of the obtained core-shell substrates with the decreased thickness shell from 29.41 nm to 5.88 nm was increased to the maximum. (Figure 4g). However, when the amounts of HAuCl_4_ increased further from 800 μL to 1000 μL, the larger size of Au NP core leads to the formation of BUT-17 shell with the thickness as thin as 1.64 nm. At this time, the SERS activity decreases, which is consistent with the reported conclusion [12]. The results showed that the modest regulation of shell thickness is to benefit of SERS performance of the whole core-shell structure.

**Figure 5:**
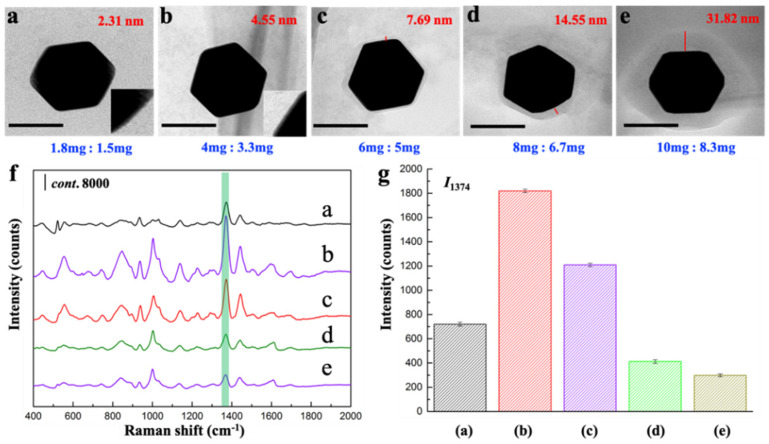
(**a**–**e**) Representative TEM images of core-shell Au@BUT-17 NP prepared with increased amount of MOF precursors (mg). Scale bar: 100 nm; (**f**) SERS spectra of thiram; and (**g**) the comparison of I_1374_ in the presence of Au@BUT-17 prepared in different amounts of MOF precursors.

Lastly, the shell thickness could also be modulated by the amount of MOF precursors including metal center (ZrCl_4_) and organic ligand (H_4_CPTTA). We fixed the ratio between ZrCl_4_ and H_4_CPTTA at 6:5, while the absolute qualities of them (mg) were controlled to (a) 1.8:1.5, (b) 4:3.3, (c) 6:5, (d) 8:6.7, and (e) 10:8.3, respectively (Table S3). The shell thickness increases continuously from 2.31 nm, 4.55 nm, 7.69 nm, 14.55 nm to 31.82 nm with the increasing amount of ZrCl_4_ and H_4_CPTTA (Figure 5a–e, Figure S5). The SERS activity increased at first but then decreased with the increasing of the thickness of MOF shell (Figure 5f,g). I_1374_ gradually increased as the shell thickness increased from 2.31 nm to 4.55 nm. However, when the shell thickness is greater than 4.55 nm, the SERS activity presented a declining trend. A shell too thick is not conducive to SERS performance, and too large a weight from the thick coating of outer MOF also gives rise to the precipitate of prepared core-shell NPs (Figure S6).

**Figure 6:**
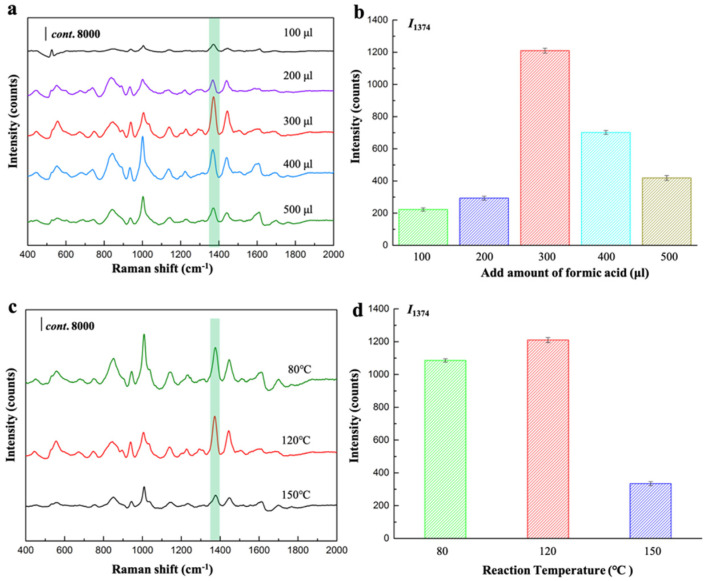
(**a**,**c**) SERS spectra of thiram; and (**b**,**d**) the comparison of I_1374_ in the presence of Au@BUT-17 prepared with the increased amount of formic acid and the reaction temperature.

In addition, attention should also be paid to the other two parameters which were also associated with the SERS performance of core-shell substrates. One is formic acid, which seems to be the raw material for the construction of MOF structures. The other is the reaction temperature, which may determinate the growth rate of MOF shell, thus resulting in the varied thickness of the formed shell. We investigated the SERS responses for thiram in the presence of core-shell Au@BUT-17 NPs prepared with the increased amount of formic acid (Table S4) and reaction temperature (Table S5), as shown in Figure 6a–d, respectively. The porous BUT-17 structure was obtained through the reaction of ZrCl_4_ and H_4_CPTTA in the presence of formic acid as competing reagent in DMF [14]. It was found that when no formic acid was added to the reaction solution, the core-shell NPs were not observed (Figure S7). Further, the quality ratio between ZrCl_4_, H_4_CPTTA, and formic acid fixed as 6:5:300 (mg/mg/μL) seems to be the optimized ingredient benefitting to the constructions of outer BUT-17. Other improper quality ratios are not in favor of the crystallization of the ordered MOF structure (Figure S8), which cannot endow the function of fixing the adsorption orientation and conformation of target molecules. This speculation was evidenced by SERS signal collection between three different tests (Figure S9). The relative standard deviation (RSD) of I_1374_ was larger than 30%, indicating the significantly unstable SERS detection.

### 3.3. Evaluate the Feasibility of the Developed Regulation Strategy

**Figure 7:**
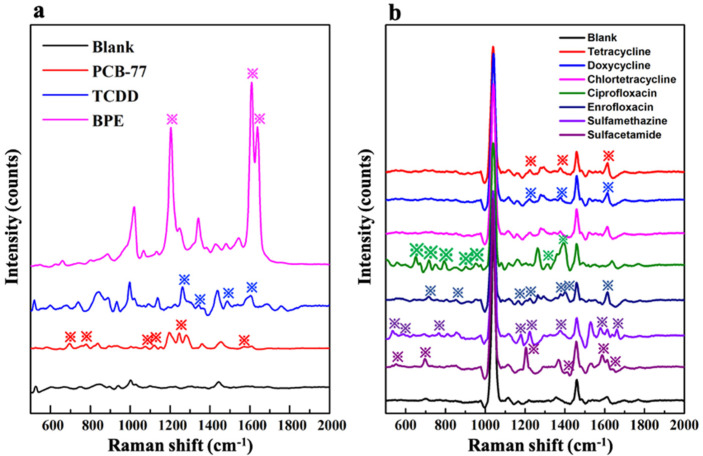
(**a**) SERS spectrum of the representative dioxin (2,3,7,8-TCDD and PCB-77) and BPE compound; (**b**) SERS spectrum of seven kinds of the familiar antibiotic residue (tetracycline, doxycycline, chlortetracycline, ciprofloxacin, enrofloxacin, sulfamethazine, and sulfacetamide) in the presence of the optimized core-shell Au@BUT-17 substrates. The characteristic Raman peaks were labeled with asterisk.

We chose two representative dioxin compounds (2,3,7,8-tetrachlorodibenzo-p-dioxin, TCDD and 3,3′,4,4′-Tetrachlorobiphenyl, PCB-77), an emerging bisphenol molecule (1,1-bis(4-hydroxyphenyl)ethane, BPE), and seven kinds of the familiar antibiotic residues (tetracycline, doxycycline, chlortetracycline, ciprofloxacin, enrofloxacin, sulfamethazine, and sulfacetamide) as the target compounds. Based on the developed regulation strategy, the core-shell Au@BUT-17 substrates were optimized to suitable shell thickness for sensitive SERS recognition of the above ten different target analytes (the synthesis conditions were supplied in Table S6). The developed Au@BUT-17 with a suitable shell thickness has demonstrated certain SERS activity towards these analytes (Figure 7a,b). However, to achieve better SERS performance based on core-shell-type substrates, further works need to be carried out, such as the selection of the MOF type, the optimum species and morphology of the MNP core.

## 4. Conclusions

In conclusion, the regulation of shell thickness is crucial for optimizing the SERS activity of the core-shell-type NMNP@MOF heterostructure towards the target compound. The control of the synthesis parameters, such as the reaction temperature, reaction time, quantity of HAuCl_4_, and the amount of MOF precursors is found to be a feasible strategy to regulate the thickness of shell in core-shell composites. When fixing the incubation time, the SERS activity of core-shell Au@BUT-17 increased at first and then decreased with the increasement of shell thickness. It was believed that this work may expand our understanding of the relationship between shell thickness and SERS activity of core-shell substrates, while providing a promising strategy for regulating the shell thickness of core-shell platform for potential trace SERS detection or enhanced catalysis.

## Data Availability

All data used to support the findings of this study are included in the article.

## Supplementary Materials

The following supporting information can be downloaded at: https://www.mdpi.com/article/10.3390/s22187039/s1, Figure S1: (a) TEM images of Au@BUT-17 structures synthesized in PVP-DMF reaction solution without ethanol; (b) TEM images of Au@BUT-17 structures synthesized in DMF-ethanol reaction solution without PVP; Figure S2: EDX elemental mapping of prepared substrates prepared in different reaction time: 25 min (a); 60 min (b); 120 min (c); 180 min (d); 240 min (e); and 300 min (f). Scale bars represent 100 nm; Figure S3: representative TEM image of as-prepared Au NPs and Au@BUT-17 with the same diameter; Figure S4: EDX elemental mapping of prepared substrates prepared with increased amount of HAuCl_4_: 200 μL (a); 400 μL (b); 600 μL (c); 800 μL (d); and 1000μL (e). Scale bars represent 100 nm; Figure S5: EDX elemental mapping of prepared substrates prepared with increased amount of MOF precursors. ZrCl_4_: H_4_CPTTA = 1.8:1.5 (a), 4:3.3 (b), 6:5 (c), 8:6.7 (d), and 10:8.3 (e). Scale bars represent 100 nm; Figure S6: compared with thinner shell (group a–d), the prepared Au@BUT-17 substrates with shell thickness of 31.82 nm (group e) precipitate partially; Figure S7: core-shell NPs were not observed when the formic acid was not added to the reaction solution; Figure S8: improper quality ratio is not favor of the crystallization of MOF structure; Figure S9: SERS spectrum of probed thiram compound (*C* = 100 ng·mL^−1^) in the presence of substrates obtained in the reaction time of 25 min. The collected spectrum from three different tests demonstrated uncontrollable SERS signal intensity (I_1374_) with relative standard deviation (RSD) up to 63.1%. Table S1: the reaction time influenced the growth of MOF shell in core-shell composites; Table S2: added amount of Au precursors indirectly change the shell thickness; Table S3: shell thickness could also be modulated by the added amount of MOF precursors including metal center (ZrCl_4_) and organic ligand (H_4_CPTTA); Table S4: formic acid is the raw material for the construction of MOF shell; Table S5: reaction temperature determinates the growth rate of MOF shell, thus resulting the varied thickness of formed shell; Table S6: the synthesis conditions of Au@BUT-17 with different MOF shell thickness for SERS detection towards ten target compounds.
