# Supplementary material for: Facile Regulation of Shell Thickness of the Au@MOF Core-Shell Composites for Highly Sensitive Surface-Enhanced Raman Scattering Sensing
Source: Sensors (Basel). 2022 Sep 17;22(18):7039. doi: 10.3390/s22187039 (PMC9504720; doi:10.3390/s22187039)
Supplement: Supplementary file 1 [file sensors-22-07039-s001.zip › sensors-1921231-supplementary.pdf]

Supplementary Information

Facile regulation of shell thickness of the Au@MOF core-shell composites for highly sensitive surface-enhanced Raman scattering sensing

Boen Li, Yaling Liu and Jie Cheng*

Institute of Quality Standards and Testing Technologies for Agro-products, Chinese Academy of Agricultural Sciences, Beijing, 100081, China

* Corresponding authors: chengjie@caas.cn

Regulation of the thickness of MOF shell in Au@BUT-17 NPs

Table S1. The reaction time influence the growth of MOF shell in core-shell composites.

NO.	Reaction time (min)	T (°C)	DMF-Ethanol (v:v)	$ZrCl_4$ (mg)	H_4CPTTA (mg)	Formic Acid (μl)	1*wt* % $HAuCl_4$ (μl)	PVP (mg)	Shell thickness (nm)
a	**25**	120	5:3	6	5	300	600	1.2	1.2
b	**60**								3.85
c	**120**								4.23
d	**180**								7.69
e	**240**								15.38
f	**300**								30.77

Table S2. Added amount of Au precursors indirectly change the shell thickness.

NO.	Reaction time (min)	T (°C)	DMF-Ethanol (v:v)	$ZrCl_4$ (mg)	H_4CPTTA (mg)	Formic Acid (μl)	1*wt* % $HAuCl_4$ (μl)	PVP (mg)	Shell thickness (nm)
a	180	120	5:3	6	5	300	**200**	**0.4**	29.41
b							**400**	**0.8**	9.6
c							**600**	**1.2**	7.69
d							**800**	**1.6**	5.88
e							**1000**	**2.0**	1.64

Table S3. Shell thickness could also be modulated by the added amount of MOF precursors including metal center ($ZrCl_4$) and organic ligand (H_4CPTTA).

NO.	Reaction time (min)	T (°C)	DMF-Ethanol (v:v)	$ZrCl_4$ (mg)	H_4CPTTA (mg)	Formic Acid (μl)	1*wt* % $HAuCl_4$ (μl)	PVP (mg)	Shell thickness (nm)
a	180	120	5:3	**1.8**	**1.5**	300	600	1.2	2.31
b				**4**	**3.3**				4.55
c				**6**	**5**				7.69
d				**8**	**6.7**				14.55
e				**10**	**8.3**				31.82

Table S4. Formic acid is the raw materials for the construction of MOF shell.

NO.	Reaction time (min)	T (°C)	DMF-Ethanol (v:v)	$ZrCl_4$ (mg)	H_4CPTTA (mg)	Formic Acid (μl)	1*wt* %HAuCl4 (μl)	PVP (mg)
a	180	120	5:3	6	5	**100**	600	1.2
b						**200**		

c						**300**		
d						**400**		
e						**500**		

Table S5. Reaction temperature determinates the growth rate of MOF shell, thus resulting the varied thickness of formed shell.

NO.	Reaction time (min)	T (°C)	DMF-Ethanol (v:v)	$ZrCl_4$ (mg)	H_4CPTTA (mg)	Formic Acid (μl)	1*wt* % $HAuCl_4$ (μl)	PVP (mg)
a	180	**80**	5:3	6	5	300	600	1.2
b		**120**						
c		**150**						

Table S6. The synthesis conditions of Au@BUT-17 with different MOF shell thickness for SERS detection towards 10 target compounds

Target	Reaction time (min)	Temperature (°C)	DMF-Ethanol (v:v)	$ZrCl_4$ (mg)	H_4CPTTA (mg)	Formic Acid (μl)	1*wt* %$HAuCl_4$ (μl)	PVP (mg)
2,3,7,8-TCDD	180	120	5:3	6	5	300	600	1.2
PCB-77	180	120	5:3	4	3.3	300	600	1.2
BPE	180	120	5:3	6	5	300	800	1.6
tetracycline	180	120	5:3	6	5	300	600	1.2
doxycycline	180	120	5:3	4	3.3	300	600	1.2
chlortetracycline	180	120	5:3	6	5	300	600	1.2
ciprofloxacin	180	120	5:3	6	5	300	600	1.2
enrofloxacin	180	120	5:3	6	5	300	600	1.2
sulfamethazine	180	120	5:3	4	3.3	300	600	1.2
sulfacetamide	180	120	5:3	6	5	300	600	1.2

Additional Figures

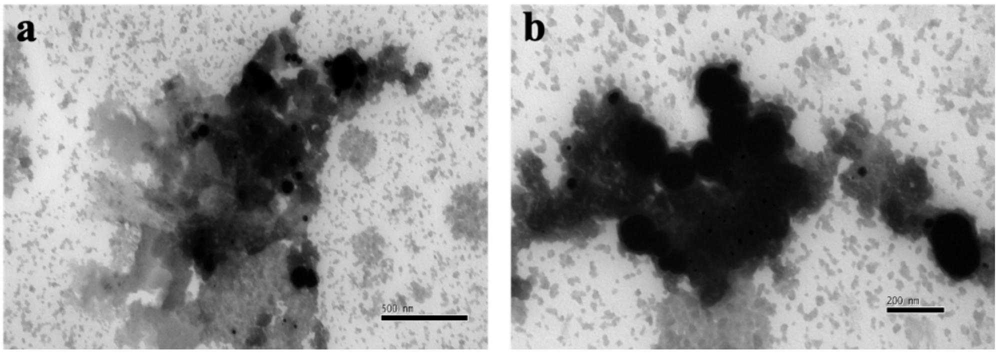

Figure S1(a) TEM images of Au@BUT-17 structures synthesized in PVP-DMF reaction solution without ethanol; (b) TEM images of Au@BUT-17 structures synthesized in DMF-ethanol reaction solution without PVP.

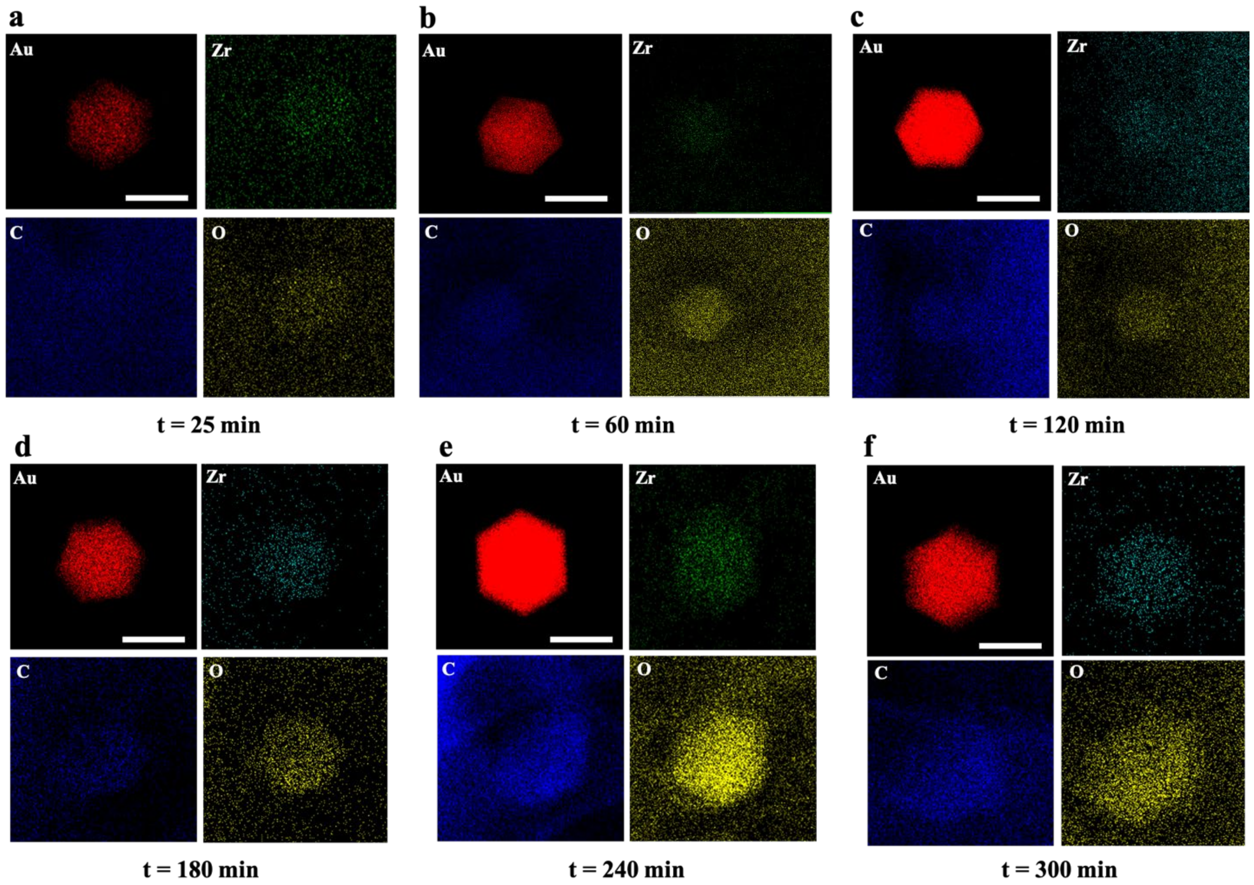

Figure S2 EDX elemental mapping of the substrates prepared in different reaction time: (a) 25 min; (b) 60 min; (c) 120 min; (d) 180 min; (e) 240 min; and (f) 300 min. Scale bars represent 100 nm.

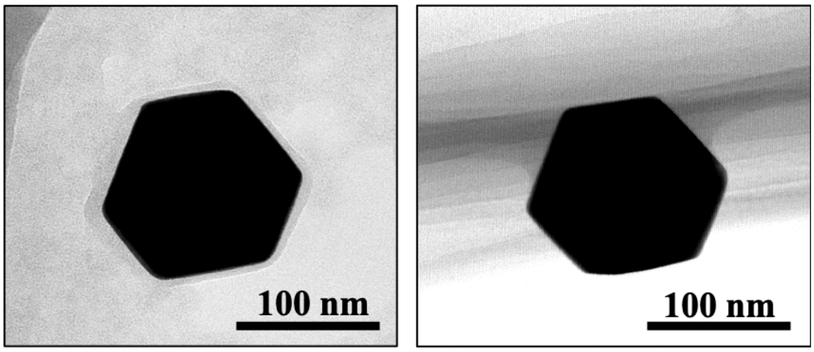

Figure S3 Representative TEM image of as-prepared Au NPs and Au@BUT-17 with the same diameter.

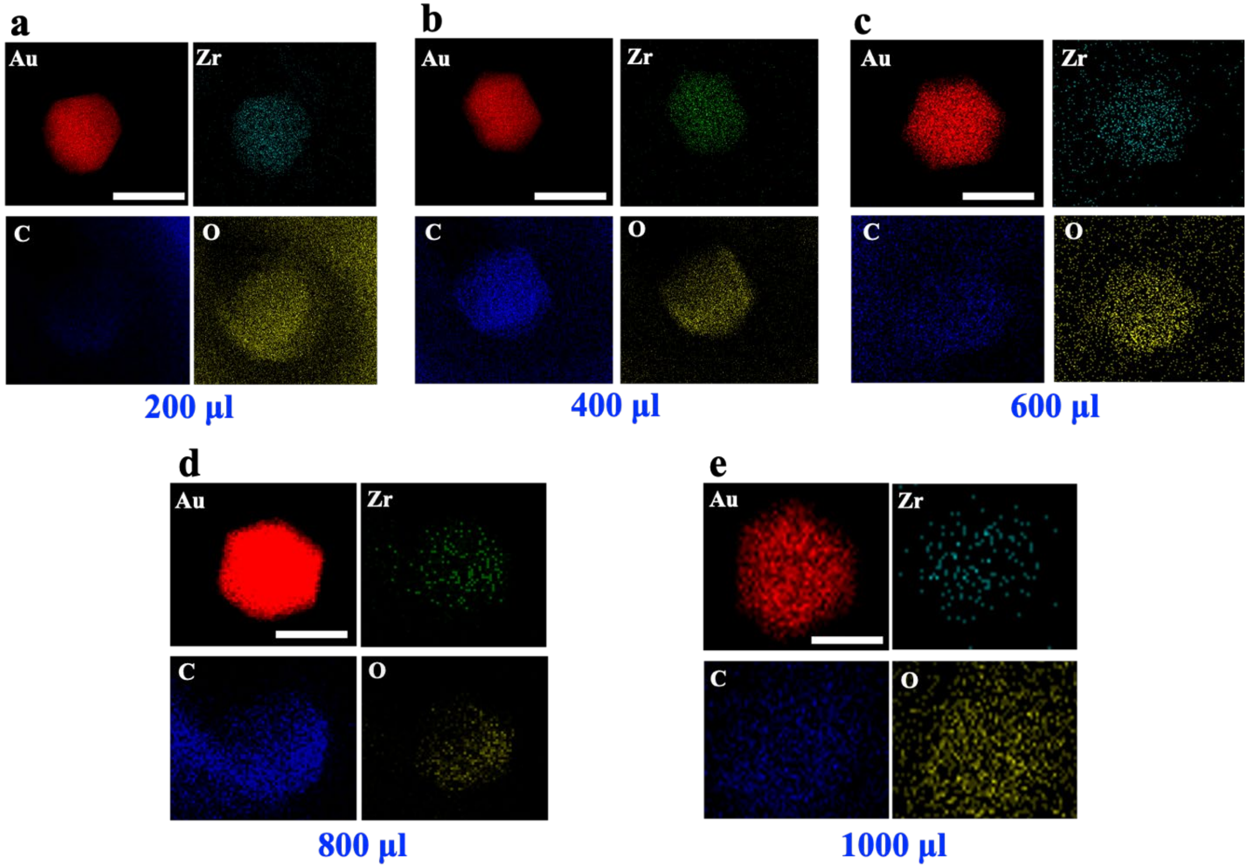

Figure S4 EDX elemental mapping of the substrates prepared with increased amount of $HAuCl_4$: (a) 200 µl; (b) 400 µl; (c) 600 µl; (d) 800 µl; and (e) 1000µl. Scale bars represent 100 nm.

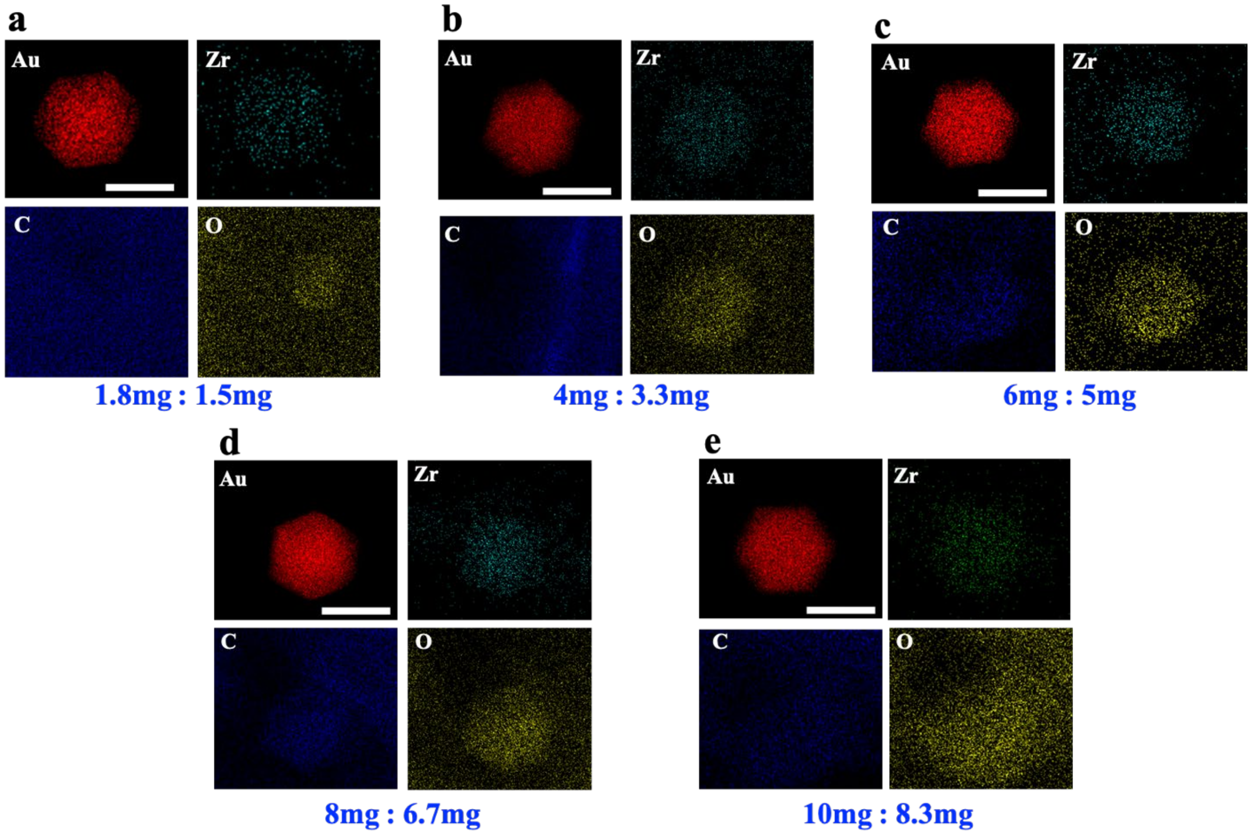

Figure S5 EDX elemental mapping of the substrates prepared with increased amount of MOF precursors. $ZrCl_4$: H_4CPTTA = (a) 1.8:1.5, (b) 4:3.3, (c) 6:5, (d) 8:6.7, and (e) 10:8.3. Scale bars represent 100 nm.

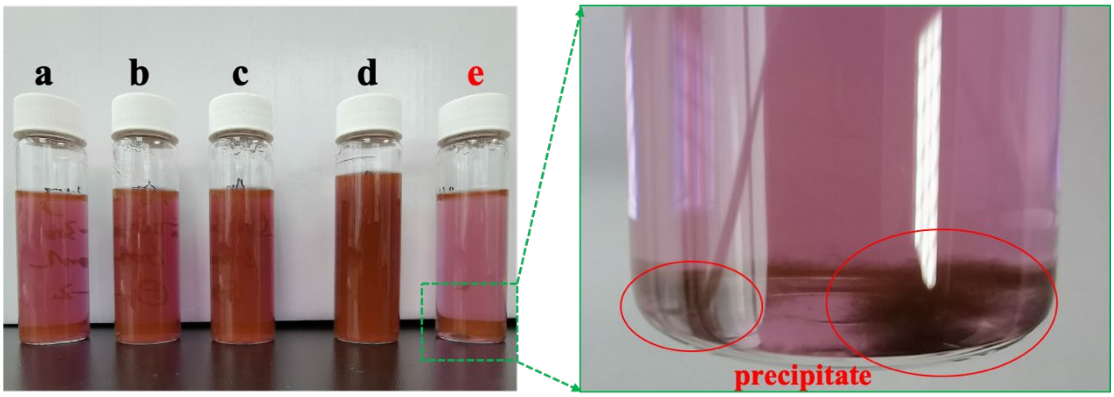

Figure S6 Compared with thinner shell (a, b, c, d), the prepared Au@BUT-17 substrates with shell thickness of 31.82 nm (e) precipitate partially.

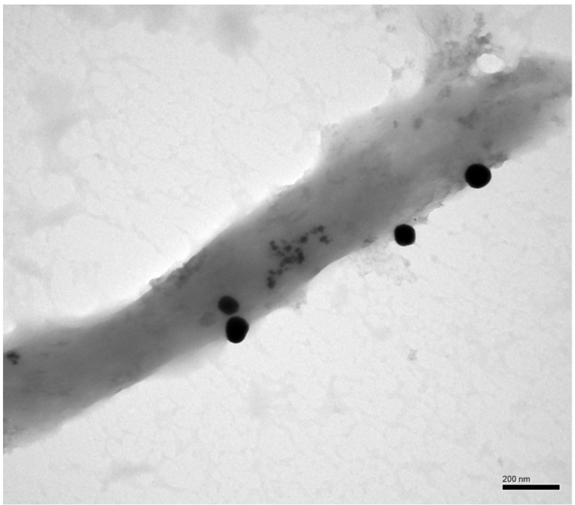

Figure S7 Core-shell NPs were not observed when the formic acid was not added to the reaction solution.

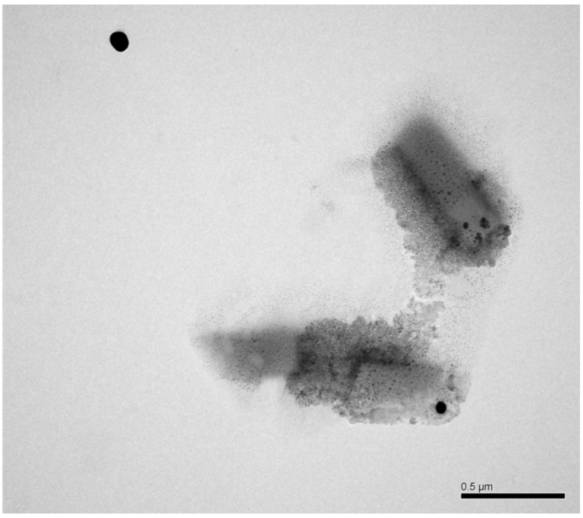

Figure S8 Improper quality ratio is not favor of the crystallization of MOF structure.

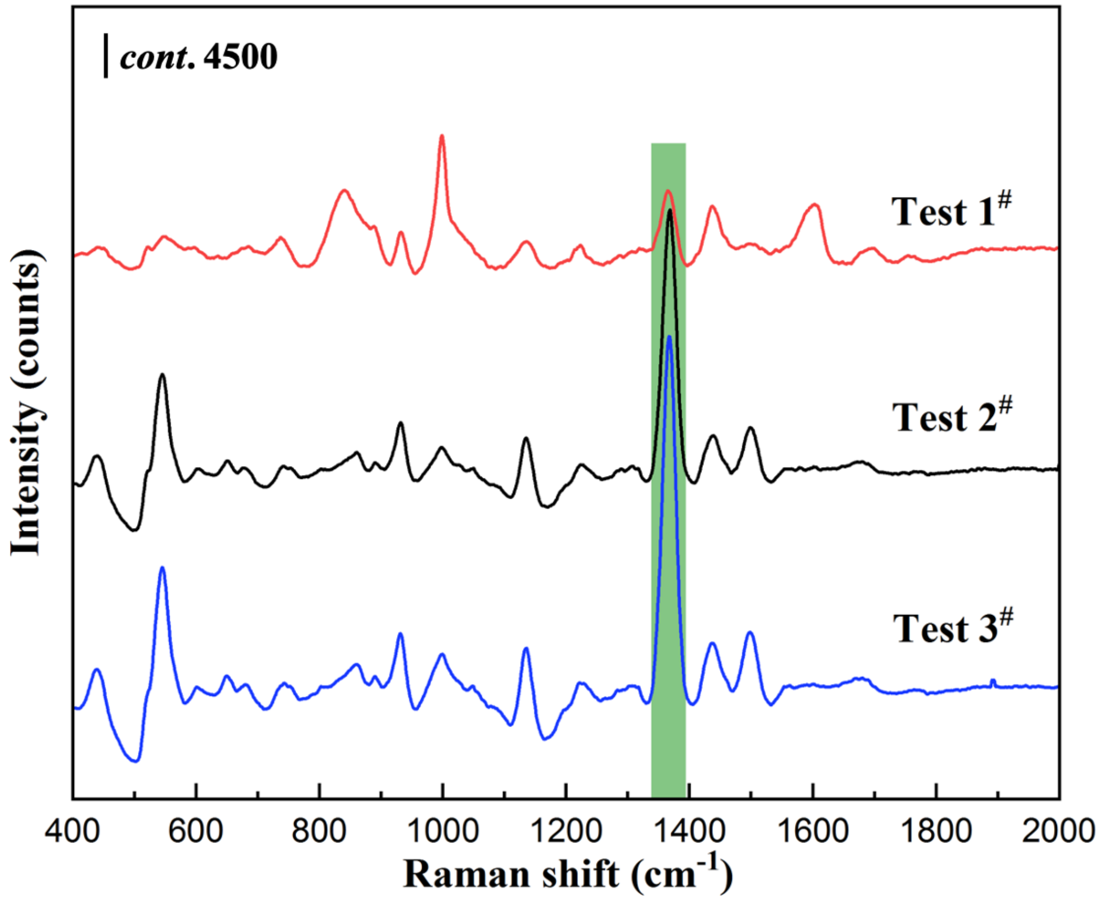

Figure S9 SERS spectrum of probed thiram compound (C = 100 ng·mL^{-1}) in the presence of substrates obtained in the reaction time of 25 min. The collected spectrum from 3 different tests demonstrated uncontrollable SERS signal intensity (I_{1374}) with relative standard deviation (RSD) up to 63.1%.
